# Potential Geographic Distribution of Brown Marmorated Stink Bug Invasion (*Halyomorpha halys*)

**DOI:** 10.1371/journal.pone.0031246

**Published:** 2012-02-21

**Authors:** Gengping Zhu, Wenjun Bu, Yubao Gao, Guoqing Liu

**Affiliations:** 1 College of Environmental Science and Engineering, Nankai University, Tianjin, China; 2 College of Life Sciences, Nankai University, Tianjin, China; University of Western Ontario, Canada

## Abstract

**Background:**

The Brown Marmorated Stink Bug (BMSB), *Halyomorpha halys* (Stål) (Hemiptera: Pentatomidae), native to Asia, is becoming an invasive species with a rapidly expanding range in North America and Europe. In the US, it is a household pest and also caused unprecedented damage to agriculture crops. Exploring its climatic limits and estimating its potential geographic distribution can provide critical information for management strategies.

**Methodology/Principals:**

We used direct climate comparisons to explore the climatic niche occupied by native and invasive populations of BMSB. Ecological niche modelings based on the native range were used to anticipate the potential distribution of BMSB worldwide. Conversely, niche models based on the introduced range were used to locate the original invasive propagates in Asia. Areas with high invasion potential were identified by two niche modeling algorithms (i.e., Maxent and GARP).

**Conclusions/Significance:**

Reduced dimensionality of environmental space improves native model transferability in the invade area. Projecting models from invasive population back to native distributional areas offers valuable information on the potential source regions of the invasive populations. Our models anticipated successfully the current disjunct distribution of BMSB in the US. The original propagates are hypothesized to have come from northern Japan or western Korea. High climate suitable areas at risk of invasion include latitudes between 30°–50° including northern Europe, northeastern North America, southern Australia and the North Island of New Zealand. Angola in Africa and Uruguay in South America also showed high climate suitability.

## Introduction

The rapid spread of invasive species that has accompanied globalization has threatened native biodiversity worldwide, and has also resulted in great economic losses [1]. As international trade increases, numbers of both accidental and intentional exotic introductions are increasing. Indeed, biological invasions have become the second most important cause of current biodiversity loss, after habitat destruction [2]. Identification of areas environmentally suitable for invasive species can offer great opportunities for preventing or slowing invasions. Recently, ecological niche modeling (ENM) has been widely used to identify the potential distributions of species [1], [3]–[5]. Based on occurrence data and environmental data sets, the ENM seeks to characterize environmental conditions suitable for the species, and then identify where those suitable environments are distributed spatially [6].

ENM analyses must be designed carefully, to reflect the fact that species' distribution manifests a complex interplay of abiotic factors, biotic factors, and dispersal constraints, that together determine distributional limits [7]–[9]. The ecological niche of a species as used herein is the set of environmental conditions under which the species can maintain populations without immigrational subsidy [10], [11]. Some recent studies have questioned the key assumption of niche conservatism during species' invasion (e.g., [12]–[14]). However, such conclusion appear artifactual [15], as they confuse differential representation of portions of the ecological niche (i.e., different “existing fundamental ecological niches” in different landscapes [16]) with genuine, evolved ecological niche difference [17]. When analyses are designed with these caveats in mind, coincidence between reciprocal prediction among native and introduced distributional areas improves markedly [17].

The Brown Marmorated Stink Bug (BMSB) *Halyomorpha halys* (Stål, 1855) (Hemiptera: Pentatomidae), is native to North and South Korea, Japan, and China. This species is becoming an invasive species showing rapid spreading in North America and Europe. The first record in America was reported in Allentown, Pennsylvania in 1996 [18]. Since then, the species has expanded its range dramatically in east America [19]. In 2005, it was reported in Vallejo, Solano County, California, facilitated by a new resident who had relocated from Pennsylvania [19]. In 2008, the species was first reported in Europe near Zürich, Switzerland [20]–[22]. By 2010, an individual was found in South Dunedin, New Zealand, apparently introduced via a used vehicle shipped from Tokyo, Japan [23]. Recently, researchers have focused on the spreading, life history and phenology, and possible control strategies for BMSB in North America [24]–[30].

In its native range, BMSB is a fruit-piercing stink bug that causes extensive damage to various fruits and soybeans, it has recently become a serious pest of apples in Japan [31]. In the US, BMSB not only represents a household pest, where it seeks winter retreats and releases unpleasant smells from stink glands when disturbed, but also has become a pest of almost unprecedented importance to agriculture, particularly in the mid-Atlantic region. Crops affected include orchard crops, vegetables, grapes, other small fruits, row crops, ornamentals, and nursery crops [32].

In this study, we explored several methods that were applied in recent studies on invasive species: climate space comparisons [33], [34], modified component space comparisons [12]–[14] and niche modeling [35]. Recent studies have suggested using pooled native and introduced distributional data to produce a consensus model of distributional potential [36]. However, this approach does not allow any independent test of model robustness, and if the ecological niche has shifted or expanded during the invasion, the pooled niche model would be overly broad to predict the distributional potential [17]. Here, we first compared niche space occupied by native and invasive BMSB populations, then evaluated native niche model transferability based two variable sets in the invade region. In the end, niche model based on the introduced range were used to locate the source region of invasion, classical niche modeling approach (i.e., Native-to-introduced ecological niche modeling) were used to explore areas of potential invasion [37].

## Methods

### Occurrence data

We assembled 552 occurrence localities of BMSB, including, localities from mainland China from the literatures and specimens records in the Institute of Entomology at Nankai University, localities from Taiwan were obtained from the Taiwan e-Learning & Digital Archives Program (http://culture.teldap.tw/culture/), localities from European from Wermelinger et al. and Wyniger and Kment [20], [22], localities from Japan and South Korea from Global Biodiversity Information Facility (http://www.gbif.org/). All of these occurrences were manifested as points of latitude and longitude. In the US, however, data were counties of known occurrence from the U. S. Department of Agriculture Animal and Plant Health Inspection Service [24], we converted these records to points by digitizing the centroid of each positive county in Arc GIS 9.2 [38] following recent suggestions [13], [14]. Localities lacking geographic coordinates were georeferenced using Google Maps, Gazetteer of China [39] or BioGeomancer (http://bg.berkeley.edu/latest/). Records with unspecified or unknown localities were deleted. The native range points covered the full known geographic range of BMSB except for North Korea due to inaccessibility of distributional data.

The native 383 occurrence points varied in spatial density due to variable sampling intensity over geography. As a result, and to avoid overemphasizing heavily on sampled area, we selected points for model calibration using a subsampling regime to reduce sampling bias and spatial autocorrelation. Following Nuñez and Medley [40], we generated models using all available occurrence points and measured spatial autocorrelation among model pseudo-residuals (1 – probability of occurrence generated by model) by calculating Moran's *I* at multiple distance classes using SAM v4.0 [41]. Significance was determined using permutation tests. A minimum distance of 335 km was detected, so we created a grid with cell dimensions of 3×3° and selected the occurrence point that close to the centroid of each grid cell. This procedure reduced the number of occurrences to 95 points used for model calibration, leaving the remaining points used for model testing. The procedure greatly reduced sampling bias and spatial autocorrelation, resulting in evenly distributed occurrence points across space [40].

### Environmental variables

Environmental dimensions in which to characterize ecological niches were selected by considering the climate, topography, habitats, and human impacts that might potentially affect BMSB distribution [31], [42]–[44]. We chose bioclimatic variables representing annual trends and extreme or limiting conditions, because many taxa are limited by environmental extremes. Variables that were highly correlated were excluded from our selection, leaving six variables summarizing aspect of temperature and precipitation that were derived from the WorldClim database [45] and three variables summarizing aspect of solar from the CliMod [46]. Topography variable represented by elevation data were also obtained from the WorldClim database. All dimensions were set at a spatial resolution of 2.5 arc-min for analysis.

Previous studies have demonstrated that using simpler and less dimensional environmental data sets improves model projections among major distributional areas [15], [17], [33]. The GLC and NDVI are global land cover types and normalized difference vegetation index respectively, these variables might have a relationship with BMSB distributions. The human footprint index is a composite summary of human influence on land surfaces, and is well known to facilitate species invasions [1]. We considered protocols of Liu et al. [1] and initially incorporated these variables into our model, however, although their incorporation improved model prediction on the native range, it did not improve model projections onto other regions. In the end, we used two sets of bioclimatic variables only, to show the reduced dimensionality effect on spatial predictions for BMSB. We first used ten bioclimatic variables representing the annual trends and extreme environmental factors of temperature, precipitation and sunshine that might impact the distribution of BMSB (Table 1). Since temperature and sunshine are two major factors that impact BMSB's distribution [31], [42], [43], and the sunshine can be related as another mean of temperature, we reduced the dimension by excluding the BIO 13, 14 and BIO 20, 21 (Table 1).

**Table 1:** Principal components analysis (PCA) of bioclimatic variables associated with occurrence of BMSB.

		Factor Loading		
Variables	Description	PC-1	PC-2	PC-3
*BIO1	Annual mean temperature	**0.93**	0.14	0.22
*BIO5	Maximum temperature of warmest month	0.45	0.48	0.56
*BIO6	Minimum temperature of coldest month	**0.92**	−0.19	0.12
*BIO12	Annual precipitation	0.66	−0.51	0.18
BIO13	Precipitation of wettest month	0.70	0.07	−0.12
BIO14	Precipitation of driest month	−0.08	−0.69	0.56
*BIO20	Annual mean radiation	−0.10	**0.88**	0.11
BIO21	Highest weekly radiation	−0.74	0.35	0.35
BIO22	Lowest weekly radiation	0.65	0.66	−0.24
*DEM	Elevation	0.14	−0.15	**−0.92**
Eigenvalue		3.84	2.38	1.76
Percentage variance		38.42	23.79	17.64
Cumulative percentage variance		38.42	62.20	79.84

*indicate the variables used in the final model construction.

Eigenvalues for the most important variables (>0.8) in PCA are in bold.

### Direct climate comparisons and Principal component analysis (PCA)

We compared climate space occupied by native and introduced populations using direct climate comparisons and principal component analysis (PCA) before ecological niche modeling, as these methods allows for quick assessment of the relative positions of populations in climate space [33], [34]. We superimposed occurrence data on the bioclimatic grids, and extracted the climate value for each occurrence using ArcGIS 9.2 [38]. The ten variables occupied by native and introduced populations were compared visually in boxplots, and statistically tested using independent sample test in SPSS 19. PCA on the correlation matrix was used to reduce dimensionality further. To facilitate data visualization among continents, occurrence points were pooled for the native Asian region (383 points), the invaded region in North America (161 points) and the invaded region in Europe (8 points).

### Modeling approach

All models were developed using a maximum entropy algorithm implemented in Maxent (version 3. 3. 3a) [47]–[49]. In exploring areas of potential invasion, another algorithm (i.e., GARP) was used (see below). Maximum entropy is a machine-learning technique that predicts species' distribution by integrating detailed environmental variables with species occurrence data. It follows the principle of maximum entropy and spreads out probability as uniformly as possible, but subject the caveat that they must match empirical information such as known presence [48]. The models were developed using the linear, quadratic, and hinge functions to avoid problem of over-fitting [49], [50]. A jack-knife procedure was used to evaluate the relative importance of each predictor variable in the model [51].

### Two variable sets comparison

To evaluate niche model predictability based on two variable sets, our models were built on native range by using occurrence points and environmental data clipped to the appropriate size then transferring them onto the US (not include Hawaii and Alaska). We used the reduced native occurrence points with an enforced distance from one another to calibrate model, leaving the remaining native occurrence data for native model evaluation. When projecting onto the US, the invasive records in the US were used for model transferability evaluation. To better exhibit the result, the logistic output of Maxent with suitability values ranging from 0 (unsuitable habitat) to 1 (optimal habitat) was used. Logistic output gives an estimate of probability of presence, it estimates probability of presence assuming that the sampling design is such that typical presence localities have probability of presence of about 0.5 [48], [49].

We used the Area Under Curve (AUC) of Receiver Operating Characteristic (ROC) plot and binary omission rate for our model comparison and evaluation. AUC is a composite measure of model performance and weights the omission error (predicted absence in areas of actual presence) and commission error (predicted presence in areas of actual absence) equally. AUC values range from 0 to 1, where 1 is a perfect fit. Useful models produce AUC values of 0.7–0.9, and models with ‘good discriminating ability’ produce AUC values above 0.9 [52]. The “area under the curve” (AUC) of the ROC plot is a threshold-independent measure of model accuracy, which juxtaposes correct and incorrect predictions over a range of thresholds. Omission rates weights mainly on omission error, our binary omission rates were calculated by the proportion of test points that were not predicted at a threshold. We plotted omission rate across the threshold spectrum of Maxent, specifically, we calculated omission rate at the increasing rate of 0.05 degrees against the total 1.0 logistic output.

### Locating source region of invasion

Although BMSB is expanding its range in the US and is far from equilibrium in Europe (i.e., not inhabiting the entire habitable area), we tentatively used “retro ecological niche modeling” approach to search the matching climate spaces occupied both by native and introduced populations and to predict its spatial distribution in Asia. These models were effectively built using invasive occurrence records and transferred onto the potential native area allow to hypothesize source regions for the invasion. We set aside 25% of these points for binary omission rates test, the remaining were used to run a ten cross-validation replicates to get a more robust result in Maxent. Six variable data set was used (Table 1). Data splitting outside (25/75) and then inside (50/50) Maxent reduced sample bias and spatial autocorrelation greatly. Cross-validation also has one big advantage over using a single training/test split that it uses all of the data for the validation [48], [49]. The cumulative output of Maxent with suitability values ranging from 0 (unsuitable habitat) to 100 (optimal habitat) was used. Cumulative output gives a prediction of suitable condition for the species above a threshold, depending on the level of predicted omission that is acceptable [48], [49]. We used the standard deviation of AUC values in ten replicates and omission rates at threshold of M10 for model evaluation. The M10 threshold assumed that a grid cell was suitable if its suitability score was more than 10, which has been suggested as an appropriate threshold [51]. We also calculated the omission rate of native occurrence to evaluate the retro model transferability.

### Exploring areas of potential invasion

To explore areas of potential invasion globally, the six variable data set was used (Table 1). We calibrated models based on native range, and transferred their prediction onto the other continents. Considering that the record in the US does not characterize the actual distribution, and the sample bias in native Asia, we used 95 occurrences of the reduced native sample for model calibration. Maxent model was first run using logistic output then rerun using cumulative output. For model evaluation, we calculated binary omission rate of the remaining occurrence at the threshold of M10. Although Maxent has appeared superior to GARP in some previous studies [4], carefully assessments of model quality showed no significant differences between the two [50]. Recent studies suggested using multiple algorithms to infer a consensus estimate of niche dimensions [51], [53]–[56]. Hence, we further used the Genetic Algorithm for Rule-set Prediction (GARP, [57]) to explore areas of potential invasion (Text S1).

## Results

### Direct climate comparisons

**Figure 1:**
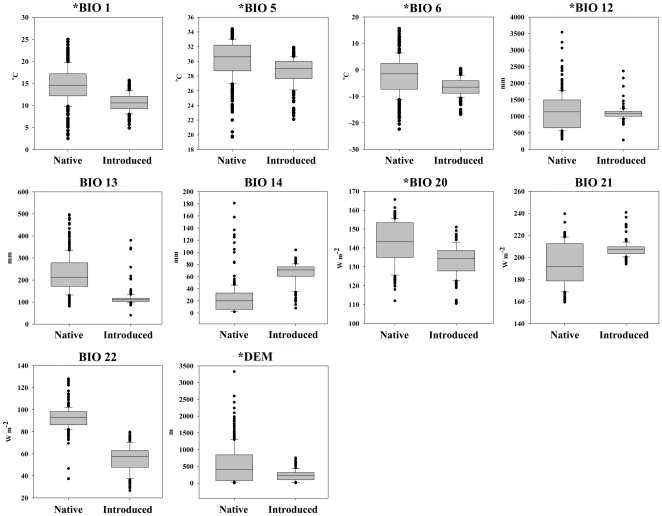
Direct comparison of BMSB occurrence-associated variables between native and introduced distributional areas. Asterisk (*) indicate variables used in the final model calibration.

Figure 1 summarized 10 climatic dimensions and their ranges among native and invasive populations of BMSB. The extreme values for the invasive population fell well within that of the native population, with the exception of precipitation in wettest month (BIO 13) and lowest weekly radiation (BIO 22), for which some invasive records fell beyond the lowest values observed on the corresponding native range (Figure 1). Introduced population occurred in areas with lower annual mean temperatures (BIO 1), lower maximum temperature of warmest month (BIO 5), lower minimum temperature of coldest month (BIO 6), lower precipitation of wettest month, lower annual mean radiation (BIO 20), lower lowest weekly radiation (BIO 22), lower elevation (DEM), and higher precipitation of driest month (BIO 14) and higher highest weekly radiation (BIO 21) (*p*<0.001). The mean annual precipitation (BIO 12) was nearly equal between native and invasive populations (Figure 1). Since BMSB is still expanding its range, incorporation of newly established populations in invaded regions might change the pattern of their distribution in climate space.

**Figure 2:**
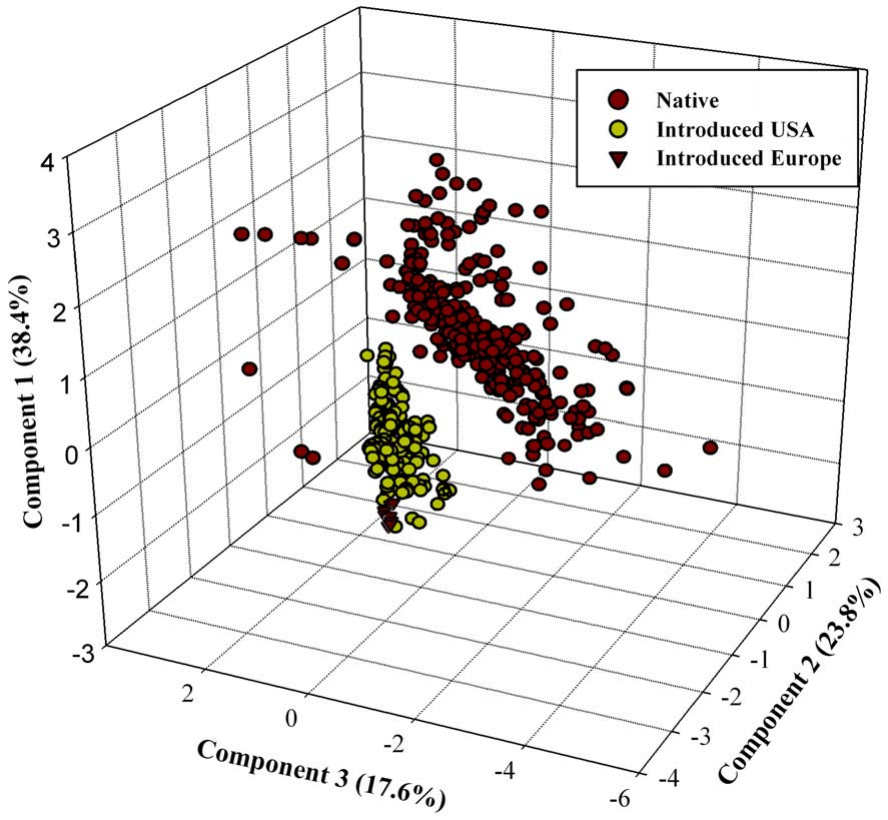
Principal component analysis of 10 variables associated with occurrences of BMSB. Symbols represent BMSB occurrences in native areas in Asia and introduced areas in the US and Europe.

Principal component analysis of the climatic data defined a climate space of reduced dimensionality that allows investigation of niche conservatism and differentiation (Figure 2). The first three components of the PCA were significant, and together explained 79.8% of the overall variance. The first component (PC-1) was closely associated with temperature while the second (PC-2) and third components (PC-3) were associated with radiation and elevation respectively (Table 1). The climate space occupied by invasive records departed from that occupied by native records with respect to component 1 and 2, but not component 3 (Figure 2). The climate space occupied by US records shifted principally along component 1, while the European records shifted along both components 1 and 2 (Figure 2). The shifting positions of invasive records in climate space might suggest that the species is undergoing change in tolerance or even niche differentiation during the invasion process. However, many alternative explanations exist: in particular, the full dimension of ecological niches may not be observed on a given range, such that these niche “differences” may rather reflect the different portions of the scare fundamental niche that are manifested on native-range versus invasive-range areas. In addition, the importance of environmental conditions can vary greatly across short distances, suggesting that the resolution of existing global environmental data sets may be too coarse to accurately describe the species' ecological niche [34].

### Environmental data sets and model comparisons

**Figure 3:**
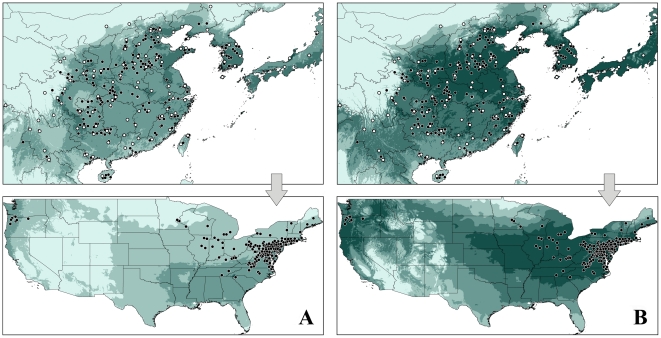
Niche models based on reduced native records and projected onto the US using Maxent. Dark green color represents high suitability, light green indicates low suitability. A: using 10 variables, B: using 6 variables, white and black dots represent the 95 occurrences for model calibration and the remaining for model evaluation.

**Figure 4:**
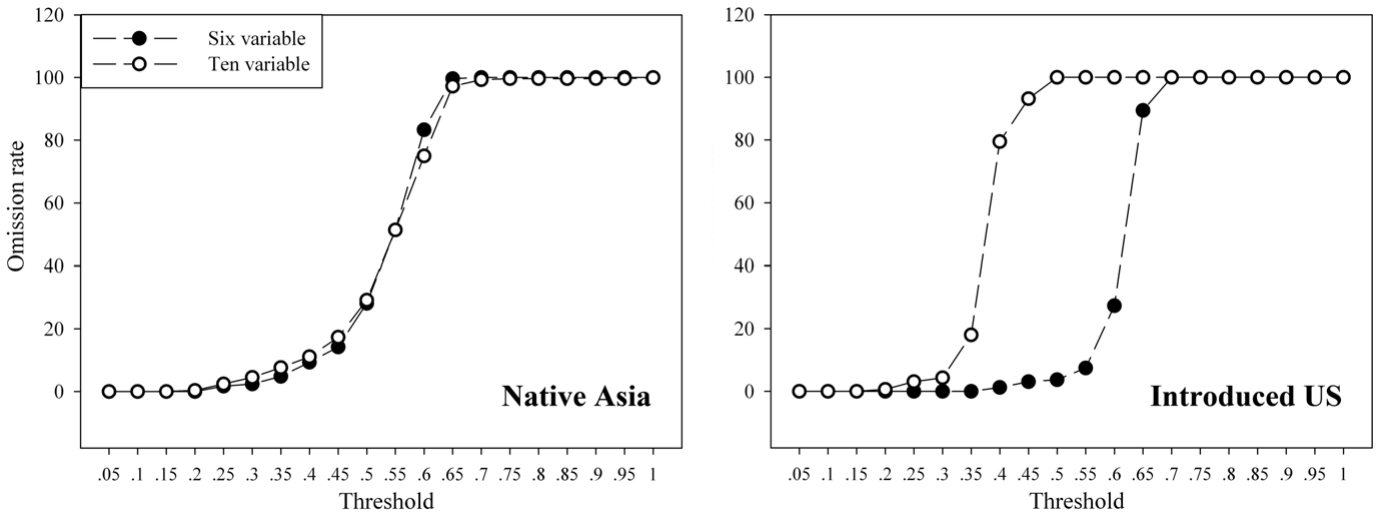
Omission rate comparison between the six- and ten-variable based models. Omission rates were plotted in native Asia models and their transferring in the US across the threshold spectrum of Maxent.

Comparing the two environmental data sets, one highly dimensional and the other simple, the simpler data set showed greatly improved model projection onto North America (Six variable: AUC = 0.894 VS Ten variables: AUC = 0.791, Figure 3), although some detail in anticipating the native range was sacrificed (Six variable: AUC = 0.765 VS Ten variables: AUC = 0.782, Figure 3). In omission rate test, no difference was observed in native model prediction based on six and ten variables, however, when transferring onto the US, model based on six variables with omission rate decreased at threshold of 0.15 to 0.7 comparing to that based on ten variables (Figure 4). Indeed, models based on both environmental data sets showed good discriminating ability compared to random prediction (Figure 3). Both model transferrings also successfully identified the current disjunct distribution of BMSM in the US, which suggests the western areas of Oregon, California and Washington state possess a similar climate space with northeast America. In six variables based model, the three west states, the northeast states, and the middle states including Kansas, Nebraska, Iowa, Missouri, Illinois, Indiana, Kentucky, Tennessee, Wisconsin, Ohio, West Virginia showed high suitability for BMSB. Preventing strategy should pay more attention to these areas to slow down the current rapid spreading.

### Locating source regions

**Figure 5:**
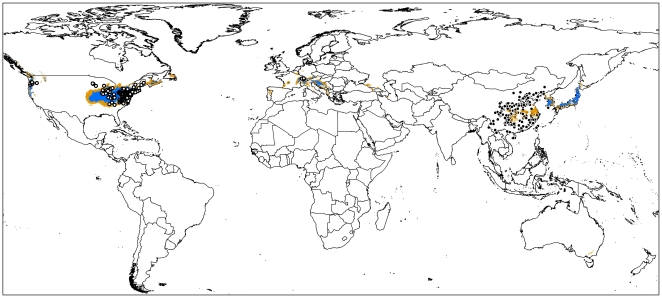
Niche model based on invasive records and transferred worldwide using Maxent. White and black dots represent occurrences of BMSB in introduced and native areas repectively, yellow areas indicate predictive probability under M10 threshold, blue areas with probability above M10 in Asia indicate the source region of invasion.

The standard deviation of 0.003 across ten crossvalidate replicates in Maxent suggests high coindence among replicate models. The omission rate at M10 was 4.65% in invaded range suggesting good model calibration, when transferring onto native areas and using native records as test data, the corresponding omission rate reached 88.68% indicating poor model transferability. However, projection of the model onto Asia identified areas of matching climate space (Figure 5): parts of Honshu in Japan, western South Korea showed high suitability of climate space that matching the introduced population. Coincidence between phylogenetic study and ecological niche modeling would provided richer evidence, but this pattern is at least interesting [13], [55], [58], [59]. The low retro model transferability also suggests the invasive population covers a portion of the climate space occupied by native population, suggesting that the invasive population is not in distributional equilibrium, or that the two distributional areas hold distinct subset of the fundamental niche space.

### Areas of potential invasion

**Figure 6:**
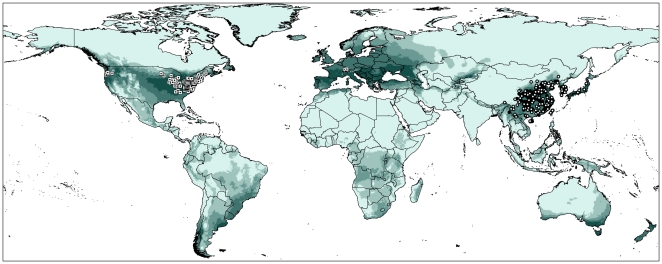
Niche model based on reduced native records and transferred worldwide using Maxent. Dark green color represents high suitability, light green indicates low suitability. White circles indicate the 95 occurrences used for model calibration, black dots and white squares represent the remaining native and invasive records used for model evaluation.

Maxent model based on the reduced 95 native points omitted 3.06% of the independent test points (total 457 points), suggesting good model performance. Projection of GARP is a little conservative comparing to Maxent (Figure 6, Figure S1). Outside of native-range areas, high suitable climate space identified by both modeling algorithms include the northeastern areas along the Pacific coast and east central states in the US in North America. Elsewhere include Uruguay and areas in southern Brazil and northern Argentina in South America, and areas around the Black Sea and the areas west to its same latitudinal range in Europe. Maxent also identified northern Europe as suitable. Northern Angola and adjacent areas of Congo and Zambia in Africa, the southeastern and southwestern Australia, and much of New Zealand also showed high climate suitability. All the areas mentioned above should pay attention to quarantine and inspection when engaging in interchanges with East Asia.

## Discussion

### Niche differentiation

The classical approach to estimating species' invasive potential calibrates models based on native range and then transferring these data onto the invade region [37]. Through assessment of species' ecological requirements and comparisons of climatic properties of native and invasive populations, we can infer the degree of niche conservatism prior to prediction [33], although not without complications. That is, the observed niche shifts may result from genuine shifts in the fundamental niche [60], or the realized niche is a subset of the fundamental niche [61], [62], observed niche difference are only interpretable as reflecting change in the fundamental niche under restrictive circumstance [33]. Specifically, the portions of the fundamental niche that are represented an actual landscape must be interpreted with considerable care.

Our results comparing niche spaces between native and invasive populations can be considered as comparing realized niches. In the US and Europe, BMSB is rapidly spreading and is far from equilibrium, while in the native China, BMSB coexists with predators and competitors, which may impact its distribution to some extent. Certainly, climate features also play a role, as well. *Erthesina fullo* (Thunberg) and *Dolycoris baccarum* (L.) are two other stinkbugs that are often reported as serious pests along with BMSB in orchards [63]–[69]. However, BMSB usually acts as the dominant species in the orchard pest community, for example, in gardens in northern Henan Province, BMSB represents about 73% of individuals, while *E. fullo* and *D. baccarum* account to about 21% and 7%, respectively [65]. Among natural enemies of BMSB in the Beijing area are 6 parasitoids and 3 predators [70], [71], the dominant natural enemy is the parasitoid *Trissolcus halyomorpha* Yang, which showed parasitism rates reaching 20%–70% (average 50%), and has been studied as a potential biological control agent [71]. However, *T. halyomorpha* does not appear to have constrained the native distribution of BMSB, as it parasitizes other stink bugs [70] and competes with other parasites [72]. So far, no effective natural enemy is available for BMSB control in Asia and the US, although many efforts explored possibilities [31].

### Dimensionality and model projections

Selection of environmental variables is very important for model calibration. Apart from biological importance that may restrict species' distributions, the resolution, extent of study range [73], and correlation among variables [37] have to be taken into consideration. Comparison of the climatic envelopes occupied by native and invasive populations offers useful information for variable selection prior to the prediction, since niches may be conserved along some environmental axes but not along others [13], [33]. We initially incorporated the GLC, NDVI, and the human footprint index into model calibration. We found that incorporation of these variables indeed improved native model prediction, however, in transferring the model onto the invaded region, the predictive ability varied with dimensionality of the environmental space. We demonstrated statistically this point by using two sets of bioclimatic variables. The reduced dimensionality improved model predictions in invaded areas greatly, although detail was lost in the native range predictions. Hence, we recommend prediction of native actual distributions using more dimensional environmental data sets, but transferring among regions using simpler models, similar recommendations have made by Peterson and Nakazawa [15], Rödder and Lötters [33], and Peterson [17].

### Source regions

Invasive species undergoing range expansion are not appropriate for testing niche conservatism by the retro modeling approach, since they haven't reach their equilibrium. However, the retro modeling can identify areas of matching climate space occupied by invasive populations in the native distributional area and potentially help us to identify the source region of invasion. The possible source region identified in our study was in accordance with the North America interception records. During 1973–1987, two interceptions of BMSB were recorded by U. S. Department of Agriculture. One was intercepted in an aircraft from Japan in 1983, and the other in baggage coming from Korea in 1984 (the original identification as *Halyomorpha picus* F. is a misidentification, as populations from Korea, Japan and China are assignable to *H. halys*, which is BMSB, *H. picus* occurs only in tropical south and southeast Asia, see Josifov & Kerzhner [74]). During 1989–1998, USDA listed eight interception records from China, Korea and Japan [18], although the details of province within countries were not known. Population genetic studies could complement these results with lineage information to identify source region much more precision [13], [55], [58], [59].

### Areas of potential invasion

Prior to inferring areas of potential invasion, one must keep in mind that the ENMs seek to identify suitable climate space for species, but without consideration of biotic interactions or dispersal ability. Many factors influence successful establishment of non-indigenous species into a community depends on existing species composition and richness, competitors, predators, food availability, human footprint, and climatic similarity, compared with the source areas [33]. Although the area predicted as suitable for a species does not mean that it can necessarily establish populations there, it does offer useful information for detecting areas of potential invasion and spread.

Many invasive species in the US have similar distribution patterns: that is populations in the northeast and a disjunct population in the northwest. Examples include the Japanese beetle (*Popillia japonica* Newman) and the Europe Chafer (*Amphimallon majale* (Razoumowsky)) [24]. This pattern reflects the fact that the northwest possesses a climate space similar to the northeast in North America. Our models successfully identified the current disjunct distribution pattern of BMSB in the US (Figures 3, 6), including successful anticipation of the specific counties from Oregon, California and Washington. Because some central states also showed high suitability for BMSB, populations may be able to form a continuous distribution in the US.

The region between latitudes 40° and 50°N in Europe showed high climate suitability, supported both by Maxent and GARP. The result of Maxent is somewhat more liberal compared to GARP, with suitable space extending north to latitude 60°N. The newly established BMSB population in Switzerland must be monitored carefully as a result. Much of New Zealand also showed high climate suitability for BMSB, although BMSB individuals newly discovered in South Dunedin have not as yet established population [23]. Attention should be paid to the high-suitability areas around the world, especially in developed areas with intensive trade activity with Japan, Korea or China must take strict quarantine inspection since commercial interchange might facilitate new invasions.

## Supporting Information

Figure S1 **Niche model based on reduced native records and transferred worldwide using GARP.** Dark green color represents high suitability, light green indicates low suitability. White circles indicate the 95 occurrences used for model calibration, black dots and white squares represent the remaining native and the invasive records used for model evaluation.
(TIF)

Text S1 GARP Protocol in exploring area of potential invasion.
(DOCX)
