# Supplementary figures and images for: Potential Geographic Distribution of Brown Marmorated Stink Bug Invasion (Halyomorpha halys)
Source: PLoS One. 2012 Feb 21;7(2):e31246. doi: 10.1371/journal.pone.0031246 (PMC3283620; doi:10.1371/journal.pone.0031246)

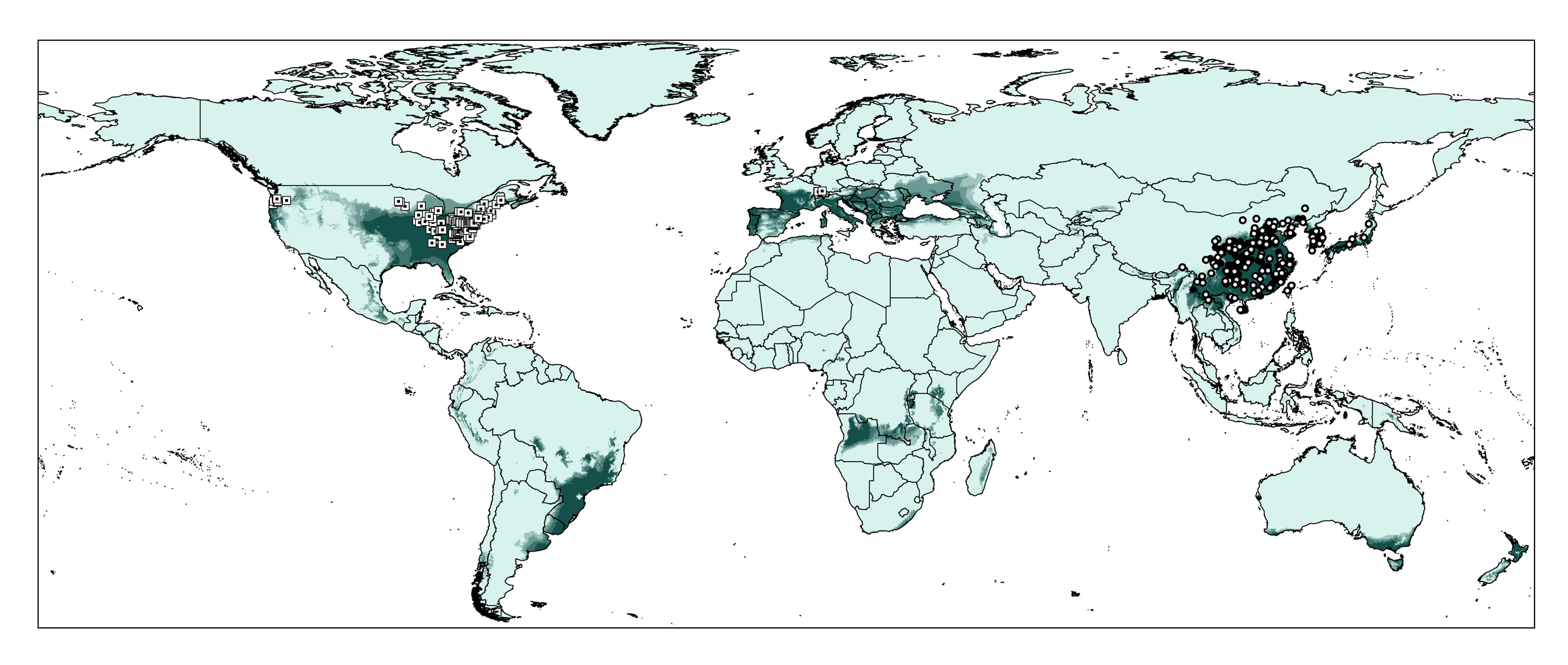

Supplement: Figure S1 — Niche model based on reduced native records and transferred worldwide using GARP. Dark green color represents high suitability, light green indicates low suitability. White circles indicate the 95 occurrences used for model calibration, black dots and white squares represent the remaining native and the invasive records used for model evaluation. (TIF) [file pone.0031246.s001.tif]
