# Supplementary material for: Potential Geographic Distribution of Brown Marmorated Stink Bug Invasion (Halyomorpha halys)
Source: PLoS One. 2012 Feb 21;7(2):e31246. doi: 10.1371/journal.pone.0031246 (PMC3283620; doi:10.1371/journal.pone.0031246)
Supplement: Text S1 — GARP Protocol in exploring area of potential invasion. (DOCX) [file pone.0031246.s002.docx]

**GARP Protocol in exploring area of potential invasion**

GARP uses an evolutionary computing genetic algorithm to search for non-random associations between environmental variables and known occurrences of species, as contrasted with environmental characteristics across the overall study area. Because model development is stochastic, and resulting models can vary in quality, we used a procedure described by Anderson *et al.* [1] to select an optimal subset of random replicate models for combination as a consensus model. In particular, we developed 100 replicate models, of these models, we retained the 20 with lowest omission error. Then, we retained the 10 models with intermediate commission error. This ‘best subset’ of models were summed to produce final predictions of potential distributions in the form of grids with values ranging from 0 (all models agree in predicting absence) to 10 (all models agree in predicting potential presence).

1. Anderson RP, Lew D, Peterson AT (2003) Evaluating predictive models of species’ distributions: criteria for selecting optimal models. Ecological Modelling 162: 211–232.
